# The Behaviours of Electromagnetic Wave Propagation in Carbon Nanotube-Layered Nanocomposites

**DOI:** 10.3390/ma19020315

**Published:** 2026-01-13

**Authors:** Ayse Nihan Basmaci, Seckin Filiz

**Affiliations:** Vocational School of Technical Sciences, Tekirdag Namık Kemal University, 59030 Tekirdag, Türkiye; sfiliz@nku.edu.tr

**Keywords:** electromagnetic wave, electromagnetic wave propagation, carbon nanotubes, nanocomposites

## Abstract

This comprehensive study delves into the intricate behaviours of electromagnetic (EM) wave propagation in a sophisticated, multilayered nanocomposite structure. The structure comprises four precisely engineered layers, each meticulously crafted from carbon nanotube (CNT) fibres arranged at specific angles and directions. These intricate arrangements not only define the structural integrity of the composite but also play a pivotal role in determining the material properties of each layer. Remarkably, when the layers are meticulously arranged at angles of [0°/90°/90°/0°] with respect to each other, the structure exhibits the highest reduced material property parameter (D*). Conversely, positioning the layers at 90-degree angles [90°/90°/90°/90°] results in the lowest reduced material property parameters, elucidating the profound influence of the arrangement patterns of the CNTs on the structural and material behaviour of the composite. Given the nanostructure nature of the composite, this study leverages the nonlocal theory to delve into the electromagnetic wave propagation frequencies (ω) and meticulously scrutinise the behaviour of transmitted and reflected electromagnetic waves within the intricate layered structure. This nanocomposite structure has been engineered as a multi-layered system, with its design grounded in the principles of nonlocal theory. Within this framework, it is revealed that, as the nonlocal parameter (η) increases, there is a discernible reduction in the frequencies (ω) of EM wave propagation through the material. This in-depth analysis aims to contribute to a fundamental understanding of electromagnetic wave propagation behaviour in complex nanocomposite structures, with potentially far-reaching implications in various technological applications.

## 1. Introduction

The discovery of carbon nanotubes has significantly advanced the fields of materials, optics, and optoelectronics technologies [1]. These nanotubes and two-dimensional nanographene represent some of the most notable nanostructures due to their remarkable length-to-width ratios. The continuous development of materials technology has not only paved the way for further exploration of these nanostructures but has also enabled in-depth studies of the propagation behaviour of electromagnetic waves in aerogels, photonic crystals, and waveguide structures utilising optical and optoelectronics technologies [2,3,4,5,6].

Composite structures, which are formed by combining at least two distinct materials, such as polymers and fibres, can be composed of multiple or single layers containing only matrix and fibre. These properties exert a significant influence on the behaviour of electromagnetic wave propagation within the structures, impacting their electrical and magnetic responses to incident electromagnetic fields [7,8,9]. The permittivity (ε) and permeability (μ) of the structure in which the electromagnetic wave propagation occurs are the two essential parameters affecting its behaviour [10,11,12,13]. Permittivity determines the ability of a material to store electrical potential energy, while permeability relates to the material’s response to a magnetic field. As an incident electromagnetic wave progresses through the composite structure, it encounters variations in permittivity and permeability, leading to either transmission or reflection into the material, thereby impacting the overall electromagnetic wave propagation behaviour [14,15,16,17]. Numerous studies have been conducted on shielding and stealth technologies based on this principle [18,19,20,21,22,23,24,25,26]. In addition, electromagnetic wave propagation in carbon-fibre-reinforced polymers, which are multilayered composite materials, is being investigated experimentally [27]. The primary objective of these studies is to engineer materials that impede the detection of electromagnetic waves by radar. In this pursuit, various factors are considered, including the layer number, density, and material property parameters such as permittivity and permeability. The development of advanced nanocomposite structures requires precise engineering to control these parameters, which influence the behaviours of electromagnetic wave propagation within the material. Notably, nanocomposites, particularly those incorporating carbon nanotubes and graphene, have garnered significant attention due to their unique electromagnetic properties and potential for stealth technology applications. Carbon nanomaterials, notably, have been subject to theoretical scrutiny. The “nonlocal theory” has been posited, demonstrating that nanomaterial atoms at the atomic scale (10^−6^ m) are influenced not only by their neighbouring atoms but also by certain other nearby atoms. This phenomenon introduces unique electromagnetic wave propagation properties at nanoscale dimensions, requiring sophisticated theoretical frameworks to understand and model the behaviour of electromagnetic waves within such materials [28,29]. Numerous studies in the literature have been predicated on this theory, advancing our understanding of the electromagnetic wave propagation behaviours of nanocomposite structures and paving the way for innovative applications in shielding and stealth technologies [30,31,32,33].

In this comprehensive study, the sophisticated behaviours of electromagnetic wave propagation within a meticulously designed tetra-layered nanocomposite structure containing carbon nanotubes (CNTs) aligned in orthotropic orientations within each layer are delved into. The investigation focuses on determining the dispersion relations of the electromagnetic waves, employing nonlocal theory and nonlocal effects within the nanocomposite structure for a thorough analysis. The research encompasses nanocomposite structures with varying layers designated as I, II, III, and IV, each accommodating CNT fibres arranged at distinct angles, including 30°, 45°, 60°, 75°, and 90°. Through rigorous calculations, the study meticulously computes the reduced material property parameters (D*) specific to this nanocomposite structure, accounting for factors including the carbon nanotube aspect ratio, orientation angle, and interlayer interactions. In this study, the reinforcing fibres within the nanocomposite are specified as carbon nanotubes (CNTs). It is assumed that the interfaces between the layers are free from any defects, ensuring ideal bonding throughout the structure. Furthermore, each individual layer is modelled as being composed exclusively of single-walled carbon nanotubes (SWCNTs), providing a uniform and well-defined nanoscale architecture for the analysis.

A pivotal aspect of the study lies in its unique focus on the electromagnetic wave propagation behaviours within a layered nanocomposite structure featuring CNT fibres arranged at specific angles within each layer, necessitating the computation of reduced material property parameters (D*), which are crucial for comprehensively understanding the effective electromagnetic wave propagation behaviours within the nanocomposite material.

## 2. Materials and Methods

**Figure 1:**
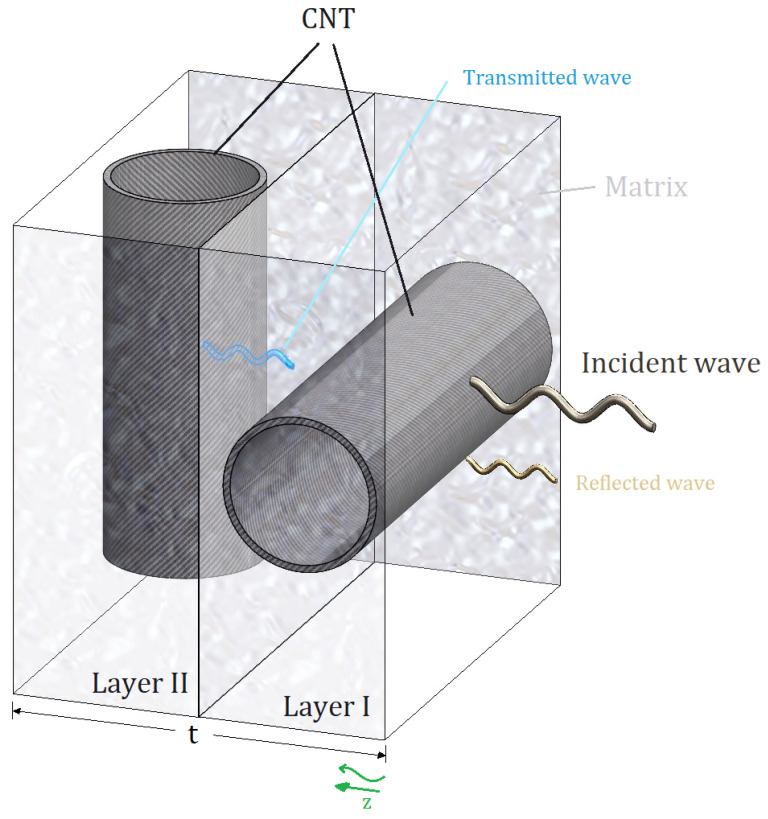
A di-layered composite structure incorporating carbon nanotube fibres within its layer.

A di-layered nanocomposite structure consisting of two layers with carbon nanotubes (CNTs) aligned in different directions within each layer is depicted in Figure 1. Specifically, there exists a 90° directional variance between the CNTs present in the structure. This 90° directional variance between the CNTs in the two layers leads to mechanical and electrical variances in the nanocomposite structure. The differing alignment of the carbon nanotubes significantly influences the material property parameters denoted as *D*, resulting in unique electromagnetic wave propagation behaviours in different directions within the structure.

In a source-free, linear, isotropic and homogenous region, the first-order Maxwell’s curl equations are [34,35]:

(1a) $$\nabla \cdot \vec{E}=0$$

(1b) $$\nabla \cdot \vec{H}=0$$

(1c) $$\nabla \times \vec{E}=-i\omega \mu \vec{H}$$

(1d) $$\nabla \times \vec{H}=i\omega \varepsilon \vec{E}$$

where µ is the permeability, ε is the permittivity, $\vec{E}$ is the electrical field, and $\vec{H}$ is the magnetic field. Using Equations (1c) and (1d), Equation (2) is derived as follows:

(2) $$\nabla \times (\nabla \times \vec{H})=\nabla (\nabla \cdot \vec{H})-\nabla^{\;2}\vec{H}=\nabla \times \left(-\mu \frac{\partial \vec{H}}{\partial t}\right)$$

By conducting a solution to Equation (2), one derives the partial differential equation that characterises the propagation of electromagnetic waves in relation to both temporal and spatial variables, as delineated:

(3) $$\frac{1}{\mu \varepsilon }\frac{\partial^{\;2}H_{z}}{{\partial \;z}^{\;2}}-\frac{\partial^{2}H_{z}}{{\partial \;t}^{2}}=0$$

The electromagnetic wave equation, initially presented in Equation (3), has been rearranged using displacement functions. In this context, i represents the imaginary unit, defined as $i=\sqrt{-1}$. The one-dimensional electromagnetic wave equation, outlined in Equation (2) is derived from Equations (1a) and (1b) in conjunction with Maxwell’s Equations (1c) and (1d). Consequently, this leads to a time-dependent electromagnetic wave equation, describing the propagation of an electromagnetic wave along the z-axis.

The electromagnetic wave propagation equation (Equation (2)) can be represented in a nonlocal format as follows [24,25]:

(4) $$D\frac{\partial^{\;2}H_{z}}{\partial z^{2}}=\left[1-{\left(e_{0}a\right)}^{2}\frac{\partial^{2}}{\partial z^{2}}\right]\frac{\partial^{2}H_{z}}{\partial t^{2}}\;\;\;and\;\;\eta :e_{0}a$$

Here, η signifies the nonlocal parameter. It is important to note that anisotropic structures possess direction-dependent properties.

Consequently, for materials characterised by anisotropic traits, the most general linear relationships regarding permittivity (ε) and permeability (μ) are represented as tensors ($\vec{\varepsilon }$ and $\vec{\mu }$) in three-dimensional (3D) form, as outlined below:

(5a) $$\overset{\to }{\varepsilon }=\left[\begin{array}{ccc} \varepsilon_{11} & \varepsilon_{12} & \varepsilon_{13}\\ \varepsilon_{21} & \varepsilon_{22} & \varepsilon_{23}\\ \varepsilon_{31} & \varepsilon_{32} & \varepsilon_{33} \end{array}\right],$$

(5b) $$\overset{\to }{\mu }=\left[\begin{array}{ccc} \mu_{11} & \mu_{12} & \mu_{13}\\ \mu_{21} & \mu_{22} & \mu_{23}\\ \mu_{31} & \mu_{32} & \mu_{33} \end{array}\right].$$

**Figure 2:**
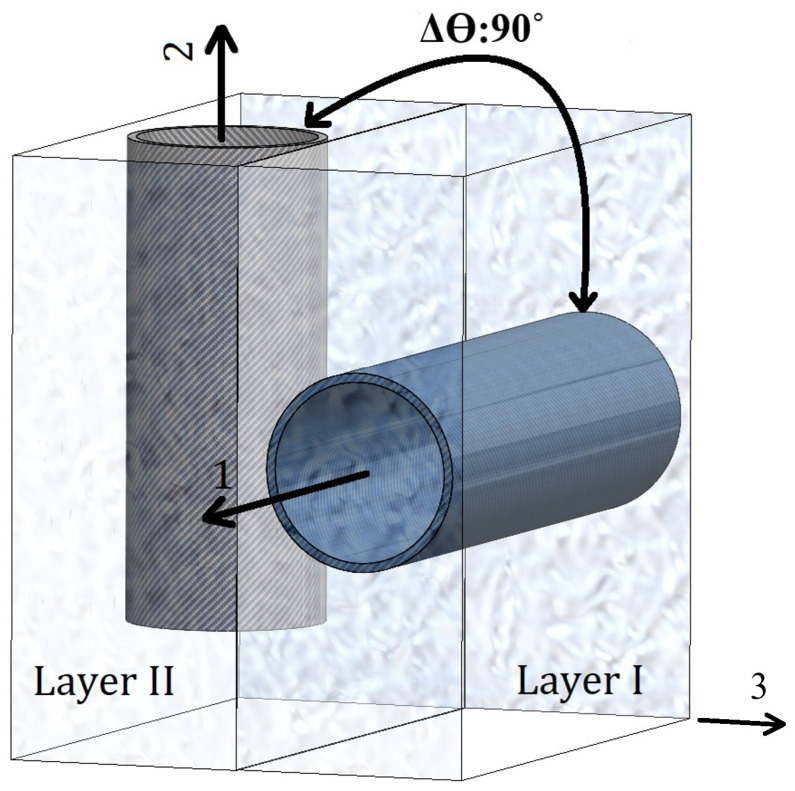
A laminate composed of CNT fibres arranged perpendicularly (Δθ: 90°) to each layer in a di-layered composite structure.

In this context, direction “1” refers to the orientation of the CNT fibre within Layer I. In Layer II, as illustrated in Figure 2, the orientation of the CNT fibre is transformed to form an angle θ in relation to their arrangement in Layer I.

As depicted in Figure 2, the thickness of each layer (t/N) is characterised on the nanoscale. Layer I is systematically oriented in the “1” direction, while the additional layers are configured at specified angles (θ) of 30°, 45°, 60°, 75° and 90°. Figure 2 shows only two-layered version of the composite structure, which can have up to four layers in total. Moreover, the nanocomposite laminate is meticulously designed to encompass 2, 3, and 4 layers, thereby ensuring the maintenance of a consistent total wall thickness. Corresponding investigations are conducted as necessary. Each layer of the composite structure displays transverse isotropy, with the “1” direction being oriented perpendicularly to the (2–3) plane.

(6) $$\varepsilon_{12}=\varepsilon_{21},\mu_{12}=\mu_{21}\;and\;indices\;3\;are\;neglected\;(2D\;lamina\;form)$$

Due to the perfect bonding between each layer, there is no intermediate region as the electromagnetic wave passes through the layers, ensuring continuity and eliminating any loss during the transition between layers. To determine the reduced material parameter in the resulting composite structure:

(7) $$\sum_{L=1}^{N}\left[D\right][\tau ](z_{L}-z_{L-1})$$

The reduced material property parameter of the composite structure D*, arranged at various angles, is determined using the transformation matrix ($\tau =\left[\begin{array}{cc} cos(\theta_{L}) & -sin(\theta_{L})\\ sin(\theta_{L}) & cos(\theta_{L}) \end{array}\right]\;\mathrm{and}\;L:1-4$) within the two-dimensional layers, as outlined in Equation (7). Following this, Equations (5a) and (5b) are substituted into Equation (4) to compute the electromagnetic wave propagation frequencies (ω). The electromagnetic wave propagation corresponding to the structure under investigation is denoted as w(z,t). As depicted in Figure 1, the propagation frequencies of the incident electromagnetic wave ($W_{I}in$), the transmitted electromagnetic wave ($W_{II}tr$), and the reflected electromagnetic wave ($W_{I}rf$) are delineated as follows:

(8a) $$w_{I}\left(z,t\right)=W_{I}e^{i\left(\omega t-k_{I}z\right)}+W_{I}e^{i\left(\omega t+k_{I}z\right)\;\;\;}\;\;\;or\;\;{\;\;\;w}_{I}\left(z,t\right)=W_{I}in+W_{I}rf,$$

(8b) $$w_{II}\left(z,t\right)=W_{II}e^{i\left(\omega t-k_{II}z\right)}\;\;\;\;\;or\;\;\;\;\;\;w_{II}\left(z,t\right)=W_{II}tr.$$

In this context, k denotes the wave number, while $W_{I}$ represents the amplitude in Layer I during the propagation of electromagnetic waves. Similarly, $W_{II}$ signifies the amplitude in Layer II. Furthermore, the reflection coefficient (Γ) and the transmission coefficient (T) for electromagnetic wave energy during propagation within the di-layered composite structure are expressed as follows:

(9a) $$\Gamma =\;{\left(\frac{k_{I}-k_{II}}{k_{I}{+k}_{II}}\right)}^{2},$$

(9b) T=1−Γ.

This study employs MathCAD 14, developed by Parametric Technology Corporation in Boston, MA, USA, as the primary computational tool to carry out the analyses. All calculations and simulations are performed using this software environment to ensure precision and reproducibility. Notably, the methodology of this research does not rely on any approximate or numerical methods for solving the governing equations; instead, exact analytical solutions are derived throughout the study.

In the context of the nanocomposite structure under investigation, particular attention is given to the reduced material property parameter values, denoted as D*, within each individual layer. These values are evaluated as functions of the fibre orientation angle within the composite, allowing for a comprehensive understanding of how anisotropy influences the system’s response. It is observed that an increase in the reduced material property parameter (D*) for any given layer leads to a corresponding increase in the electromagnetic frequencies (ω) of the system. This relationship suggests that microscale material tailoring can effectively manipulate EM wave propagation characteristics.

Conversely, the effect of the nonlocal parameter, represented as η, exhibits an inverse correlation with the electromagnetic wave frequency. As the nonlocal parameter increases, the frequency of the electromagnetic wave decreases. This finding highlights the significance of nonlocal effects—such as scale-dependent phenomena and interactions beyond the immediate neighbourhood of a material point—in determining the overall dynamic behaviour of the nanocomposite structure.

Given these relationships, it becomes evident that both the reduced material property parameter (D*) and the nonlocal parameter (η) play crucial roles in dictating the electromagnetic waveforms propagating through the nanocomposite. The interplay among these parameters directly influences the propagation characteristics of EM waves, including amplitude, phase velocity, and frequency content. These effects will be discussed in greater detail and further illustrated in the following section, where the resulting electromagnetic waveforms and their dependence on the aforementioned parameters are comprehensively analysed.

## 3. Results and Discussion

The material property parameter values have been analysed for two distinct cases, herein referred to as Case I and Case II, as detailed in Table 1, Table 2, Table 3 and Table 4. Literature indicates that the material property parameter in nanotubes typically ranges from 0.01 to 0.5 [19,20]. Accordingly, ${\left(\mu \epsilon \right)}_{\;\;11}=16,{\left(\mu \epsilon \right)}_{\;\;22}=1\;and\;{\left(\mu \epsilon \right)}_{\;\;12}=0.5$ are characteristic of Case I, whereas ${\left(\mu \epsilon \right)}_{\;\;11}=16,{\left(\mu \epsilon \right)}_{\;\;22}=7\;and\;{\left(\mu \epsilon \right)}_{\;\;12}=4$ characterise Case II. In the case in which ${\left(\mu \epsilon \right)}_{\;\;12}=0$, the reduced material property parameter (D*) in the “1” direction converges to D* = 1/16 = 0.0625. Conversely, for carbon nanotube fibre alignment in the “2” direction (*θ*_L_ = 90°), it is anticipated that this parameter will approach D* = 0. The results of the analyses for Cases I and II, which explore composite structures formed by fibre carbon nanotubes arranged at various angles, are detailed in Table 1, Table 2, Table 3 and Table 4. Specifically, Table 1 presents the reduced material property parameters (D*) for the composite structure consisting of a single layer. A careful examination of the data provided in Table 1, Table 2, Table 3 and Table 4 reveals that the reduced material property parameters vary within the range of 0.03 to 0.1.

Table 2 provides the reduced material property parameters of the composite structure consisting of two layers.

Table 3 presents the reduced material property parameters for a composite structure consisting of three layers.

Table 4 provides the reduced material property parameters of the composite structure consisting of four layers.

**Figure 3:**
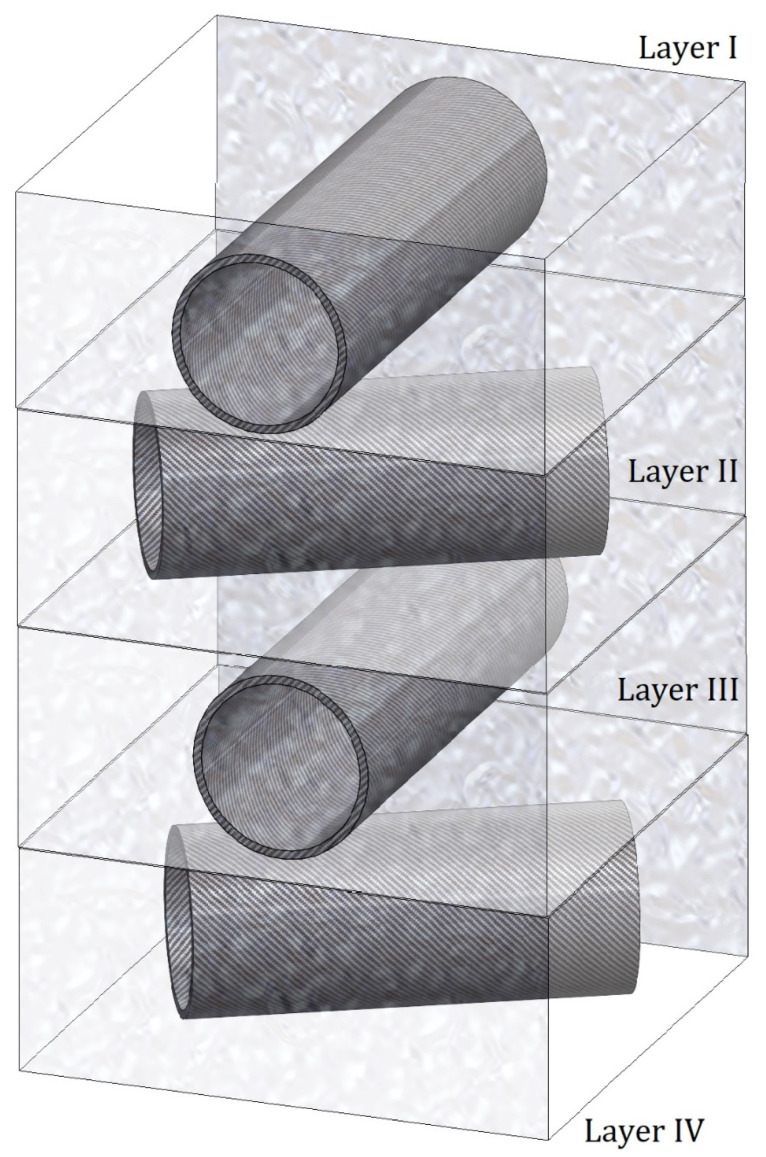
A tetra-layered nanocomposite structure is arranged with an angular pattern of [0°/60°/0°/60°] between the layers in Case I.

The tetra-layered nanocomposite structure, as depicted in Figure 3, showcases a precise angular arrangement of its constituent layers: [0°/60°/0°/60°]. This unique configuration plays a pivotal role in the analysis, particularly due to the incorporation of the 60° angle. This angle is thoughtfully chosen because it occupies a critical position between 0° and 90°, enabling a balanced and comprehensive perspective on electromagnetic wave propagation.

To fully understand the impact of each layer within the composite, a meticulous examination of its interactions and contributions is undertaken. This study investigates explicitly two configurations in Case I: the simpler mono-layered nanocomposite structure and the more intricate tetra-layered structure characterised by the specified angular arrangement. This comparative analysis seeks to elucidate the differences in electromagnetic wave behaviour between the two configurations. The findings and metrics detailed in Table 1, Table 2, Table 3 and Table 4 provide an extensive evaluation of the composite structure, treating it as an integrated whole rather than as isolated layers. Through an in-depth analysis of the gathered data, it has been established that the tetra-layered arrangement with the angular sequence of [0°/60°/0°/60°] demonstrates the second-highest reduced material property parameters (D*) among the configurations studied.

This outcome underscores the significance of layer orientation and its influence on the overall properties and performance of the nanocomposite, making it a valuable subject of study.

Moreover, the implications of this research highlight the potential applications of such a composite in various fields that depend heavily on the efficiency of electromagnetic wave propagation, including telecommunications, advanced materials science, and electromagnetic shielding.

In addition, for Case I involving the analysis of two di-layered composite structures characterised by an angular arrangement of [0°/60°] between the layers, the reduced material property parameter, denoted as D*, is calculated to be D*: 0.08157. This specific configuration highlights the interaction effects between the two layers, which contribute to the composite’s overall material properties. On the other hand, for Case I of the tetra-layered composite structure, which features a more complex angular arrangement of [0°/60°/0°/60°], the reduced material property parameter D* is found to be D*: 0.08261. This layered configuration allows for additional interaction between the layers, leading to a slight increase in the D* value compared to the di-layered structure.

These results indicate an intriguing trend: as the number of layers in the composite structure increases, the calculated reduced material property parameters appear to converge. This suggests that the composite’s mechanical performance may stabilise with additional layers, potentially leading to improved predictability in the behaviour of multi-layered composites under various loading conditions. Such insights are critical for the design and optimisation of advanced composite materials used in engineering applications. In analysing layered nanocomposite structures—including monolayer, dilayer, triolayer, and tetralayered configurations—it is essential to recognise that all these constructions are designed to maintain a constant overall thickness denoted by t. For illustrative purposes, in the tetralayered configuration, the total thickness is evenly distributed, with each of the four layers having a thickness of t/4. This uniformity ensures meaningful comparisons between different layering schemes.

The assessment of mechanical and material properties relies on Equation (7) and its associated transfer matrix methodology. Through this approach, the study identifies the highest value of the reduced material property parameter (D*) in the tetralayered structure, particularly when the orientation of the layers follows the angular sequence [0°/90°/90°/0°]. Specifically, under the conditions defined as Case II, the maximum D* value recorded is 0.010992 for the tetralayered arrangement [0°/90°/90°/0°]. Comparatively, for the triolayered structure, a similarly elevated D* value of 0.010833 is achieved when the angular arrangement is [0°/90°/0°]. These findings underscore the influence of both the number of layers and their orientations on the resulting material properties. Given the observed trends and in order to systematically explore the effects of multilayer stacking and angular arrangements, this study has set the maximum number of layers at four. This decision enables a comprehensive investigation into the property enhancements achievable through advanced layering strategies in nanocomposite materials.

This analysis examines layered nanocomposite structures—monolayer, dilayer, triolayer, and tetralayered—each designed to maintain a constant overall thickness, t. In the tetralayered configuration, each layer has a thickness of t/4, allowing for consistent comparison across different schemes. Material property parameters are evaluated using Equation (7) and the transfer matrix method. The highest reduced material property parameter (D*) is observed in the tetralayered structure with the [0°/90°/90°/0°] orientation, reaching 0.010992 under Case II conditions. The trio-layered structure achieves a comparable D* value of 0.010833 to that of the [0°/90°/0°] arrangement. These results highlight the impact of both layer count and orientation on material properties. To systematically investigate these effects, the study limits the maximum number of layers to four.

In Figure 3, the propagation of electromagnetic waves through the distinct layers of the nanocomposite structure is examined by evaluating each layer independently. This methodology enhances the understanding of electromagnetic wave behaviour as it traverses the various layers of the composite material. The analysis is founded on the reduced material property parameters, D*, outlined in Table 1, which provide essential insights into the characteristics of each layer.

These parameters serve as reference values for the respective layers, allowing for the observation of variations in wave dynamics due to differing material compositions. As the electromagnetic wave progresses through the four-layer nanocomposite structure, the data presented in Table 1 delineates the differences in transmission and reflection characteristics across each individual layer. Table 4 integrates these findings, facilitating an evaluation of the entire tetra-layer nanocomposite structure as a cohesive entity. The strong interlayer bonding diminishes the presence of intermediate regions, thereby reducing intra-material losses and ensuring that the electromagnetic wave propagates with minimal attenuation or distortion as it transitions from one layer to another.

**Figure 4:**
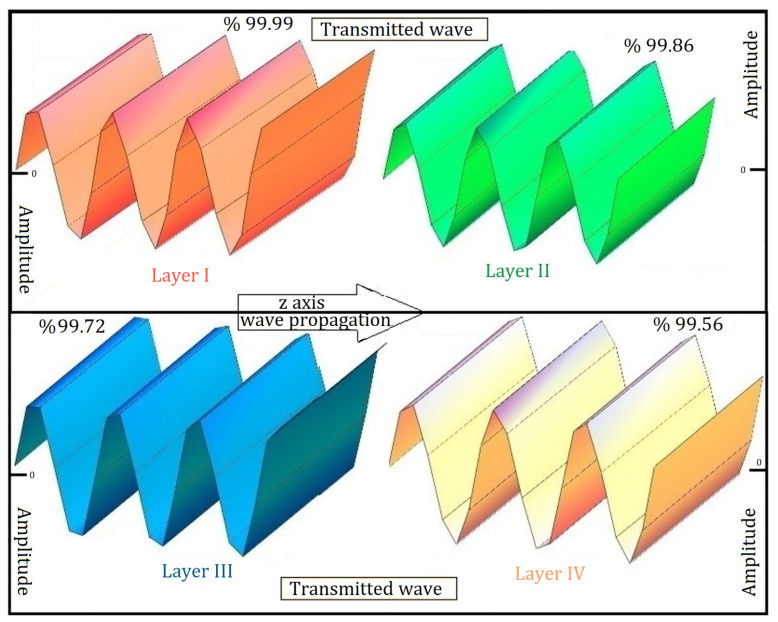
In Case I, transmitted electromagnetic waveforms are analysed in the tetra-layered composite structure with layer angles of [0°/60°/0°/60°].

Furthermore, Figure 4 offers a comprehensive analysis of the waveform of the electromagnetic wave as it transmits through each layer, progressing from Layer I to Layer IV of the composite structure as depicted in Figure 4. This illustration captures the amplitude and phase variations in the electromagnetic wave, emphasising the influence of each layer’s material properties on the overall wave behaviour. Through this detailed analysis, significant insights into the transmission mechanisms inherent to the targeted nanocomposite architecture are achieved.

**Figure 5:**
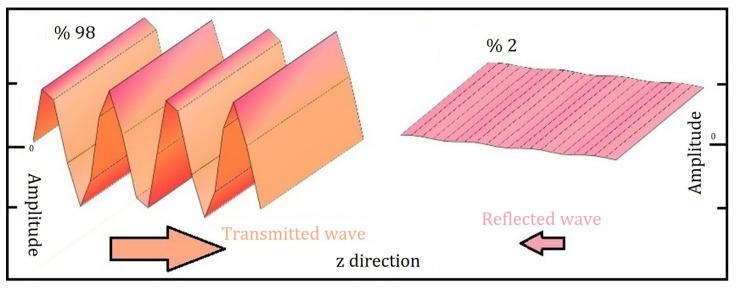
In Case I, transmitted and reflected electromagnetic waveforms are analysed in the tetra-layered composite structure with layer angles of [0°/60°/0°/60°].

The waveform of the electromagnetic wave emitted from the nanocomposite structure depicted in Figure 5 is thoroughly analysed. This particular structure consists of four distinct layers, each contributing to its overall behaviour. In this analysis, it is observed that the amplitude of the transmitted wave reaching the external environment is an impressive 98%. This high percentage indicates that the majority of the wave successfully passes through the composite material. Conversely, only 2% of the wave is reflected back, suggesting that the layers are highly effective in minimising reflection. The examination of the structure is approached from a holistic perspective, focusing on its overall performance on a macro scale rather than on individual layers. This comprehensive view is essential for understanding the efficiency of the composite in transmitting electromagnetic waves.

**Figure 6:**
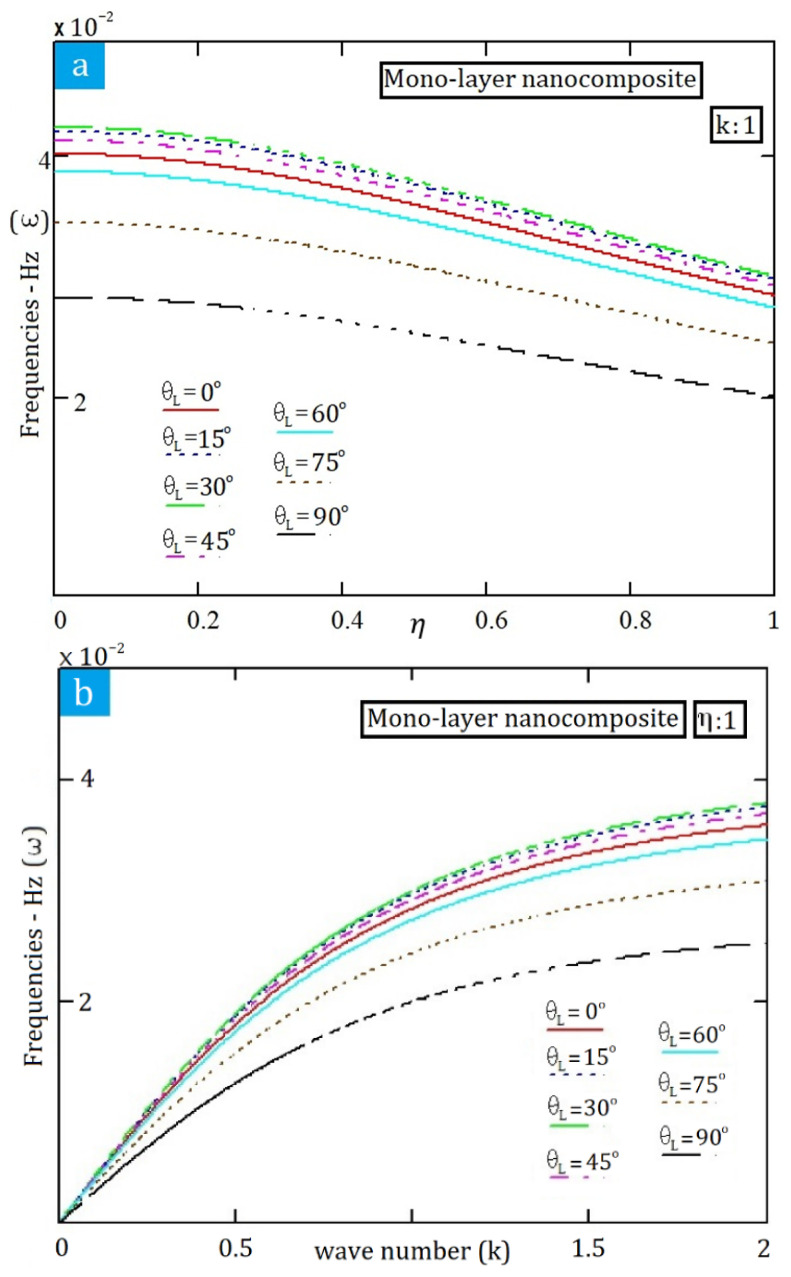
Case I: Mono-layered nanocomposite. (**a**) Impact of the nonlocal parameter (η) on electromagnetic wave propagation frequencies (ω), (**b**) Dispersion relation.

In the detailed analysis of nanocomposite structures, this study focuses on obtaining the frequency values associated with the propagation of electromagnetic waves when the nonlocal parameter is not equal to zero (η≠0). These values have been derived from the equation presented in Equation (4), which articulates the correlation between nonlocal effects and wave propagation characteristics. The results are illustrated in Figure 6, which provides a comprehensive representation of the influence of the nonlocal parameter on the propagation behaviour of electromagnetic waves within the nanocomposite material. An examination of the trends depicted in this figure allows for a deeper understanding of how variations in the nonlocal parameter affect the efficiency and characteristics of electromagnetic wave propagation. This insight is essential for optimising the design and application of such materials in advanced technological domains.

In Figure 6, a comprehensive analysis reveals that the lowest observed frequencies of electromagnetic wave propagation (ω) occur specifically at an angle $\theta_{L}$ = 90°. This finding is particularly important as it indicates a key relationship between the angle of incidence and the efficiency of wave propagation within the studied medium. Furthermore, as illustrated in Figure 6a, there is a well-defined trend showing that the values of electromagnetic wave propagation frequency (ω) exhibit a decrease in response to an increase in the nonlocal parameter (η). This trend signifies that as the nonlocal parameter escalates, the influence of spatial dispersion becomes more pronounced, thereby affecting the propagation dynamics.

Additionally, Figure 6b presents data showing an upward trend in the wave number within the nanocomposite structure. This increase in wave number suggests enhanced interaction mechanisms at play within the composite, likely due to its unique microstructural characteristics. Interestingly, once the wave number reaches approximately 2 or exceeds this threshold, the values of ω exhibit a consistent horizontal trend. This behaviour indicates a possible stabilisation of the electromagnetic properties of the waves being studied, potentially signalling a shift in the governing physical phenomena as the wave number continues to vary. Such insights may have significant implications for the design and optimisation of nanocomposite materials in applications involving electromagnetic wave propagation.

**Figure 7:**
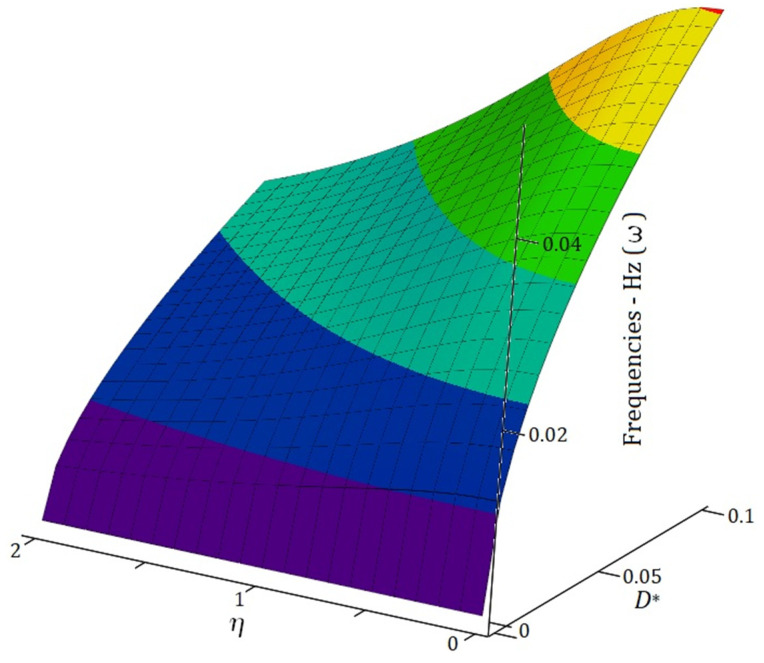
Effects of reduced material property parameters (D*) and nonlocal parameters (η) on electromagnetic wave propagation frequencies (ω) in nanocomposite structures, with a constant wave number (k:1) across all cases.

Figure 7 presents the influence of the reduced material property parameter (D*) and the nonlocal parameter (η) on electromagnetic frequencies. To maintain consistency with Case I and Case II, D* values are selected up to 0.1, and the nonlocal constant (η) ranges from 0 to 2 to facilitate a comprehensive analysis of its impact. The effect of (η) becomes pronounced as its value approaches 2. The figure demonstrates that at higher η values, electromagnetic wave propagation frequencies decrease significantly. Additionally, for D* values near 0.1, an increase in D* corresponds to an increase in electromagnetic wave propagation frequencies.

**Figure 8:**
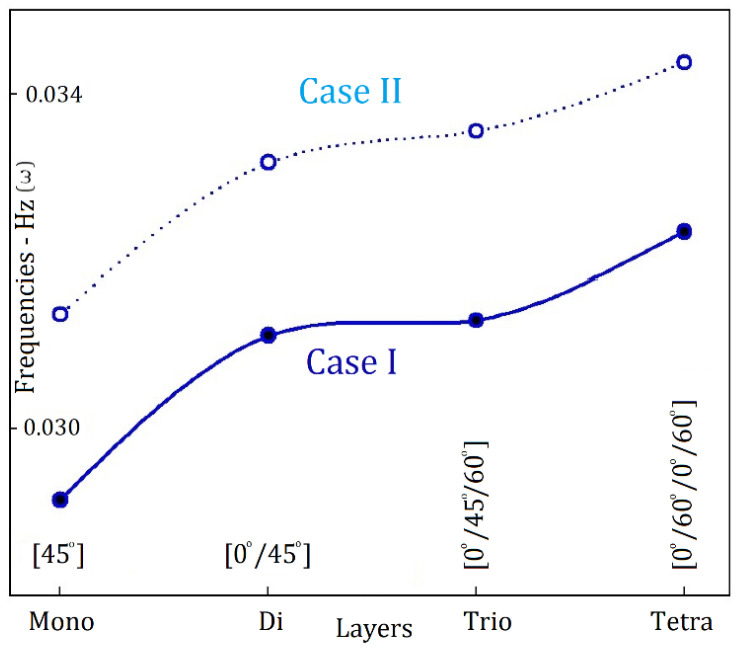
Cases I and II: Frequencies of electromagnetic wave propagation (ω) within the nanocomposites when (η:1).

In Figure 8, the data demonstrate that the frequencies of electromagnetic wave propagation, denoted as (ω), are markedly higher in Case II than in the other cases evaluated. This observation indicates an enhanced efficiency of electromagnetic wave propagation under the specific parameters associated with Case II. Furthermore, it is evident that an increase in the number of layers corresponds with a distinct upward trend in the values of ω. This correlation emphasises the substantial influence of the layered structure on the characteristics of electromagnetic wave propagation, suggesting that the addition of layers significantly improves the frequency response.

## 4. Conclusions

This study examines the intricate behaviour of electromagnetic wave propagation within multilayer nanocomposite structures, which are increasingly relevant in various technological domains, including telecommunications, sensing, and advanced materials engineering. The primary focus of this investigation is on nanocomposite structures consisting of a varying number of layers, specifically ranging from one to four, each configured with different angular arrangements. These unique orientations play a critical role in influencing the electromagnetic wave propagation behaviours of the composites. Carbon nanotubes, which serve as the reinforcing fibres due to their exceptional mechanical and electrical characteristics, form the foundation of these structures, thereby enhancing the overall performance of the composite materials. Utilising calculated reduced material property parameters, the study investigates the propagation of electromagnetic waves along the z-axis in each layer of the nanocomposites. The findings reveal significant variations in performance based on the angular configurations of the layers. Notably, the lowest reduced material property parameters correspond to the arrangement designated as [90°/90°/90°/90°], while the highest parameters are observed with a more advantageous configuration of [0°/90°/90°/0°]. This highlights the profound impact of layer orientation on the electromagnetic characteristics of the materials. Additionally, the research identifies a noteworthy correlation between the number of layers in the composite structure and the frequency of electromagnetic wave propagation. As the number of layers increases, there is a corresponding rise in the frequencies associated with electromagnetic wave propagation, emphasising the potential for multilayer designs to effectively manipulate electromagnetic wave propagation behaviour and enhance functionality in practical applications. To gain a comprehensive understanding of electromagnetic wave propagation dynamics, the study meticulously analyses several critical parameters, including transmission, reflection, and amplitude of the electromagnetic waves. This analysis provides essential insights into how varying frequencies influence the behaviour of electromagnetic waves as they propagate through the composite materials. Moreover, the study offers an in-depth examination of the electromagnetic waveforms, analysing their shapes and behaviours during traversal of the multilayered structures. This level of detail is crucial for advancing the comprehension of the underlying principles governing wave interactions with complex materials.

In conclusion, this research paves the way for future investigations to expand upon these findings. There is substantial potential for exploring electromagnetic wave propagation in layered nanocomposites with diverse material property parameters in each layer, as well as in two-dimensional and three-dimensional nanocomposite configurations. Such studies may significantly enrich the understanding of composite material behaviour and enhance their applicability across various advanced engineering technologies.

## Figures and Tables

**Table 1:** Mono-layered composite.

Mono-Layered Composite	$D^{*}$						
CNT angles ($\theta_{L}$)	$\theta_{L}$ = 0°	$\theta_{L}$ = 15°	$\theta_{L}$ = 30°	$\theta_{L}$ = 45°	$\theta_{L}$ = 60°	$\theta_{L}$ = 75°	$\theta_{L}$ = 90°
Case I	0.06344	0.0694	0.07064	0.06706	0.05892	0.04675	0.0314
Case II	0.07063	0.07839	0.80813	0.07772	0.06934	0.05623	0.03928

**Table 2:** Di-layered composite.

Di-Layered Composite	$D^{*}$							
CNT angles ($\theta_{L}$)	I = 90° II = 90°	I = 0° II = 0°	I = 0° II = 15°	I = 0° II = 30°	I = 0° II = 45°	I = 0° II = 60°	I = 0° II = 75°	I = 0° II = 90°
Case I	0.0314	0.06344	0.06758	0.07185	0.07645	0.08157	0.08754	0.09484
Case II	0.03928	0.07063	0.07581	0.08116	0.08691	0.09331	0.10078	0.10992

**Table 3:** Trio-layered composite.

Trio-Layered Composite	$D^{*}$							
CNT angles ($\theta_{L}$)	I = 90° II = 90° III = 90°	I = 0° II = 0° III = 0°	I = 0° II = 15° III = 30°	I = 0° II = 30° III = 45°	I = 0° II = 45° III = 60°	I = 0° II = 60° III = 75°	I = 0° II = 90° III = 0°	I = 90° II = 0° III = 90°
Case I	0.0314	0.06344	0.07102	0.0747	0.07736	0.07866	0.09497	0.07575
Case II	0.03928	0.07063	0.08022	0.08511	0.0889	0.09126	0.10833	0.08952

**Table 4:** Tetra-layered composite.

Tetra-Layered Composite	$D^{*}$							
CNT angles ($\theta_{L}$)	I = 90° II = 90° III = 90° IV = 90°	I = 0° II = 0° III = 0° IV = 0°	I = 0° II = 15° III = 30° IV = 45°	I = 0° II = 30° III = 45° IV = 60°	I = 0° II = 60° III = 0° IV = 60°	I = 0° II = 90° III = 90° IV = 0°	I = 0° II = 60° III = 75° IV = 90°	I = 90° II = 45° III = 60° IV = 75°
Case I	0.0314	0.06344	0.07375	0.0757	0.08261	0.09735	0.07217	0.05565
Case II	0.03928	0.07063	0.08384	0.08689	0.09331	0.10992	0.08476	0.06612

## Data Availability

The raw data supporting the conclusions of this article will be made available by the authors on request.

## References

[1] Iijima S. (1991). Helical microtubules of graphitic carbon. Nature.

[2] Rumyantsev V.V., Paladyan Y.A., Fedorov K.V., Gumenyk K.V. (2022). Specifics of electromagnetic wave propagation through a non-ideal 1D photonic crystal. Phys. Rev. B Condens. Matter.

[3] Ye Z., Wang K., Li X., Yang J. (2022). Preparation and characterization of ferrite/carbon aerogel composites for electromagnetic wave absorbing materials. J. Alloys Compd..

[4] Karakilinc O.O., Dinleyici M.S. (2015). Design of dual-mode dual-band photonic crystal bandpass filters for terahertz communication applications. Microw. Opt. Technol. Lett..

[5] Basmaci A.N. (2020). Characteristics of electromagnetic wave propagation in a segmented photonic waveguide. J. Optoelectron. Adv. Mater..

[6] Duman C., Kaburcuk F.A. (2019). A numerical study of ZnO random lasers using FDTD method. Optik.

[7] Tzeng J.T., Hsieh K.-T. (2020). Electromagnetic analysis of composite structures subjected to transient magnetic field. J. Compos. Mater..

[8] Singh A.P., Gupta B.K., Mishra M., Govind P.S., Chandra A., Mathur R.B., Dhawan S.K. (2013). Multiwalled carbon nanotube/cement composites with exceptional electromagnetic interference shielding properties. Carbon.

[9] Munalli D., Dimitrakis G., Chronopoulos D., Greedy S., Long A. (2019). Electromagnetic shielding effectiveness of carbon fibre reinforced composites. Compos. Part B-Eng..

[10] Lu X., Li X., Zhu W., Xu H. (2022). Construction of embedded heterostructures in biomass-derived carbon frameworks for enhancing electromagnetic wave absorption. Carbon.

[11] Zheng X., Wang H., Wen H., Guo J. (2020). Propagation characteristics of electromagnetic waves in mine roadways. J. Appl. Geophys..

[12] Fan Y., Yang D., Mei H., Xiao S., Yao Y., Cheng L.Z. (2022). Tuning SiC nanowires interphase to improve the mechanical and electromagnetic wave absorbtion properties of SiCf/SiCnw/Si_3_N_4_ composites. J. Alloys Compd..

[13] Shahid M.U., Ghaffar A., Majeed A.S., Khan Y. (2021). Propagation of electromagnetic waves in graphene-wrapped cylindrical waveguides filled with magnetized plasma. Optik.

[14] Deng G., Yang Y., Zhou Q., Lei Y., Yue L., Yang T. (2022). Lightweight and broadband electromagnetic wave absorbing foamed cement-based composites incorporated with hybrid dielectric fibers. Constr. Build. Mater..

[15] Feng L., Li W., Wang Y. (2022). Broadband electromagnetic wave absorbing metamaterial based on FeSiAl alloy. J. Magn. Magn. Mater..

[16] Zhu Y., Guan X., Yang Z. (2021). One-pot synthesis of Carbon nanotube reinforces graphene aerogels and their applications in electromagnetic wave attenuation. J. Phys. Chem. Solids.

[17] Liu Z., Wang Y., Jia Z., Ling M., Yan Y., Chai L., Du H., Wu G. (2020). In situ constructed honeycomb-like NiFeO_4_@Ni@C composites as efficient electromagnetic wave absorber. J. Colloid Interface Sci..

[18] Lee D.W., Kim H., Moon J.H., Jeong J.-H., Sim H.J., Kim B.J., Hyeon J.S., Baughman R.H., Kim S.J. (2019). Orthogonal pattern of spinnable multiwall carbon nanotubes for electromagnetic interference shielding effectiveness. Carbon.

[19] Zhao H., Han X., Han M., Zhang L., Xu P. (2010). Preparation and electromagnetic properties of multiwalled carbon nanotubes/Ni composites by γ-irridation technique. Mater. Sci. Eng. B.

[20] Deng L., Han M. (2007). Microwave absorbing performances of multiwalled carbon nanotube composites with negative permeability. Appl. Phys. Lett..

[21] Ni L., Chen S., Jiang X., Luo Y., Zou H., Liu P. (2022). Anisotropic electromagnetic wave absorption performance of Polyimide/multi-walled carbon nanotubes composite aerogels with aligned slit-like channels structure. Compos. Part A-Appl. Sci. Manuf..

[22] Long F., Wang L., Rehman S.U., Zhang J., Shen S., Wei B.M., Zhang W., Hu Y., Liang T. (2022). Double shell structured MnFe_2_O_4_ @FeO/C derived from MnFe_2_O_4_ @ZIF-8 for electromagnetic wave absortion. J. Alloys Compd..

[23] Kumar N., Somay S. (2022). Multiwall carbon nanotube enhance the invisibility effect from radar. Results Phys..

[24] Kuksin A.V., Gerasimenko A.Y., Shaman Y.P., Kitsyuk E.P., Shamanaev A.A., Sysa A.V., Eganova E.M., Slepchenkov M.M., Poliakov M.V., Pavlov A.A. (2024). Improving the emission properties of graphene-carbon nanotube hybrid nanostructures through functionalization with BaO nanoparticles and laser treatment. Appl. Surf. Sci..

[25] Kallumottakkal M., Hussein M.I., Haik Y., Latef T.B.A. (2021). Functionalized-CNT polymer composite for microwave and electromagnetic shilelding. Polymers.

[26] Gerasimenko A.Y., Kuksin A.V., Shaman Y.P., Kitsyuk E.P., Fedorova Y.O., Sysa A.V., Pavlov A.A., Gluckhova O.E. (2021). Electrically conductive networks from hybrids of carbon nanotubes and graphene created by laser radiation. Nanomaterials.

[27] Zhou J., Li Y., Zhang M., Xu E., Yang T. (2021). Effect of lay-up configuration on the microwave absorption properties of carbon fiber reinforced polymer composite materials. Mater. Today Commun..

[28] Eringen A.C. (1983). On differential equations of nonlocal elasticity and solutions of screw dislocation and surface waves. J. Appl. Phys..

[29] Hajdo L.E., Eringen A.C. (1979). Application of nonlocal theory to electromagnetic dispersion. Int. J. Eng. Sci..

[30] Mikki S. (2020). Theory of electromagnetic radiation in nonlocal metamaterials-Part I: Foundations. Prog. Electromagn. Res. B.

[31] Mikki S. (2020). Theory of electromagnetic radiation in nonlocal metamaterials-Part II: Applications. Prog. Electromagn. Res. B.

[32] Keller O., Pedersen J.H. (1989). A nonlocal description of the dispersion relation and energy flow associated with surface electromagnetic waves on metals. Scatt. Diffr..

[33] Basmaci A.N. (2021). Behaviors of electromagnetic wave propagation in double-walled carbon nanotubes. Materials.

[34] Pozar D.M. (2012). Microwave Engineering.

[35] Cheng D.K. (2013). Field and Wave Electromagnetics.

